# Digitalising mental health care: Practical recommendations from the European Psychiatric Association

**DOI:** 10.1192/j.eurpsy.2023.2466

**Published:** 2023-12-13

**Authors:** Janos L. Kalman, Gerrit Burkhardt, Jerzy Samochowiec, Christian Gebhard, Geert Dom, Miriam John, Ozge Kilic, Tamas Kurimay, Lars Lien, Meryam Schouler-Ocak, Diego Palao Vidal, Jan Wiser, Wolfgang Gaebel, Umberto Volpe, Peter Falkai

**Affiliations:** 1 Institute of Psychiatric Phenomics and Genomics (IPPG), University Hospital, LMU Munich, Munich, Germany; 2 Department of Psychiatry and Psychotherapy, LMU University Hospital, Munich, Germany; 3 Department of Psychiatry, Pomeranian Medical University, Szczecin, Poland; 4 MGZ – Medizinisch Genetisches Zentrum, Munich, Germany; 5 Collaborative Antwerp Psychiatric Research Institute (CAPRI), University of Antwerp, Antwerp, Belgium; 6 Department of Psychiatry, Bezmialem Vakıf University Faculty of Medicine, Istanbul, Turkey; 7 North-Buda Saint John Central Hospital, Buda Family Centred Mental Health Centre, Department of Psychiatry and Psychiatric Rehabilitation, Teaching Department of Semmelweis University, Budapest, Hungary; 8 National Advisory Unit on Concurrent Substance Abuse and Mental Health Disorders, Innlandet Hospital Trust, Hamar, Norway, Faculty of Social and Health Sciences, Inland Norway University of Applied Sciences, Elverum, Norway; 9 Psychiatric University Clinic of Charité at St. Hedwig Hospital, Berlin, Germany; 10 Mental Health Service, Parc Taulí University Hospital, Unitat Mixta de Neurociència Traslacional I3PT-INc-UAB, Sabadell, Spain; 11 Department of Psychiatry and Forensic Medicine, Autonomous University of Barcelona, Cerdanyola del Vallès, Spain; 12 Centro de Investigación Biomédica en Red de Salud Mental (CIBERSAM), Madrid, Spain; 13 CNWL NHS Foundation Trust, London, UK; 14 WHO Collaborating Centre DEU-131, VR-Klinikum Düsseldorf, Department of Psychiatry and Psychotherapy, Heinrich-Heine-Universität Düsseldorf, Düsseldorf, Germany; 15 Unit of Clinical Psychiatry, Department of Clinical Neurosciences/DIMSC, School of Medicine, Università Politecnica delle Marche, Ancona, Italy

**Keywords:** digital mental health, digital mental health interventions, digital phenotyping, European Psychiatric Association, telepsychiatry

## Abstract

The digitalisation of mental health care is expected to improve the accessibility and quality of specialised treatment services and introduce innovative methods to study, assess, and monitor mental health disorders. In this narrative review and practical recommendation of the European Psychiatric Association (EPA), we aim to help healthcare providers and policymakers to navigate this rapidly evolving field. We provide an overview of the current scientific and implementation status across two major domains of digitalisation: i) *digital mental health interventions* and ii) *digital phenotyping*, discuss the potential of each domain to improve the accessibility and outcomes of mental health services, and highlight current challenges faced by researchers, clinicians, and service users. Furthermore, we make several recommendations meant to foster the widespread adoption of evidence-based digital solutions for mental health care in the member states of the EPA. To realise the vision of a digitalised, patient-centred, and data-driven mental health ecosystem, a number of implementation challenges must be considered and addressed, spanning from human, technical, ethical–legal, and economic barriers. The list of priority areas and action points our expert panel has identified could serve as a playbook for this process.

## Introduction

Mental health disorders are the leading causes of disability worldwide, accounting for around 13% of the global burden of disease. Their socio-economic impact and associated costs are predicted to increase further in the next decades [1, 2]. Between 35 and 50% of patients with severe mental disorders receive no treatment, and there is a great geographic variation in the type and quality of available services [1]. Many believe that the digitalisation of mental health care could improve the accessibility, attractiveness, quality, cost-effectiveness, and precision of mental health services [3]. Furthermore, by generating data and translational knowledge, digitalisation could fuel the research of mental health disorders and deliver new, innovative, and personalised strategies for preventing, detecting, and managing these often chronic and debilitating conditions.

**Figure 1.**
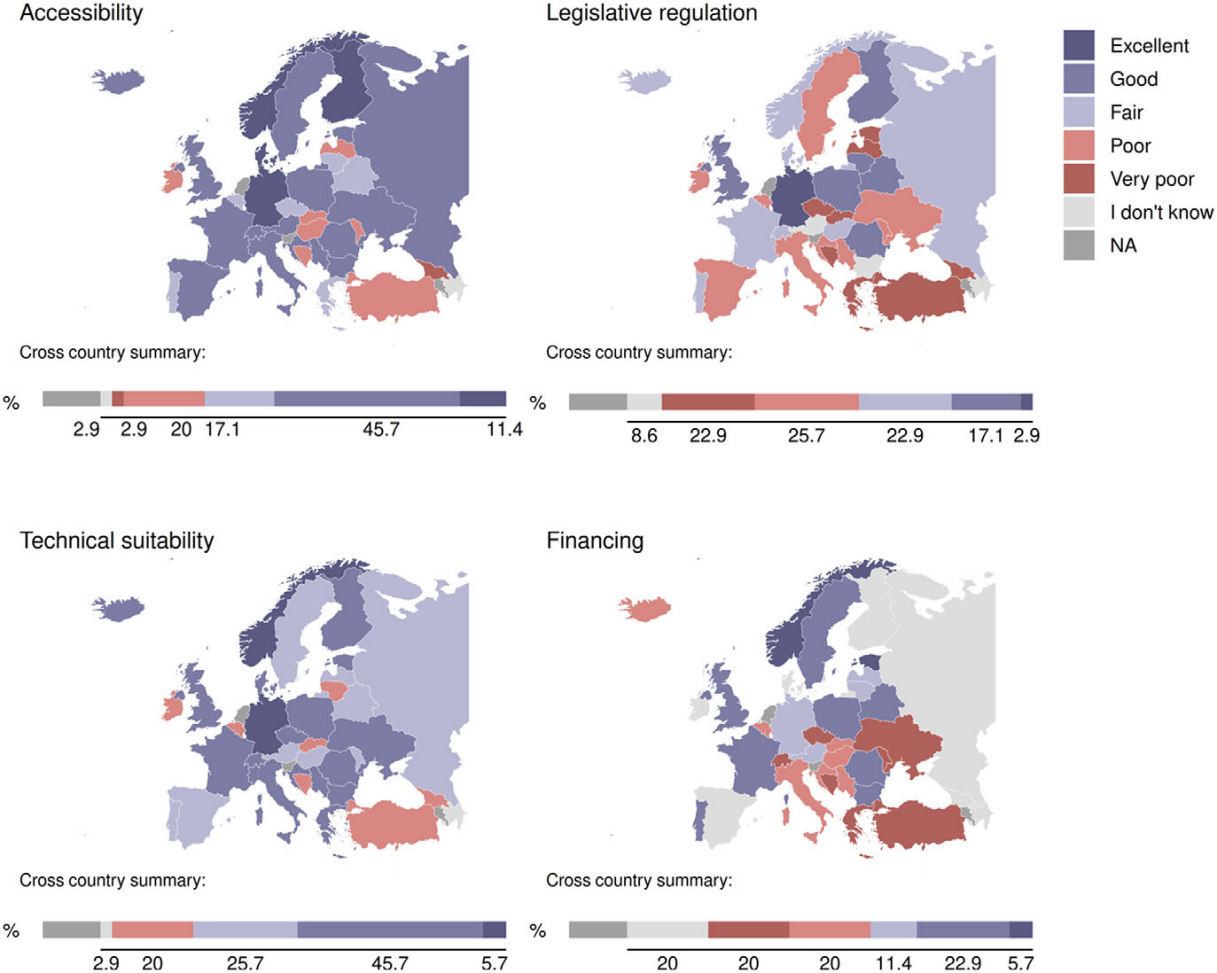
Status quo of accessibility, technical suitability, and legislative and financial regulation of telepsychiatry in the European Psychiatric Association member states. A task force of the EPA’s National Psychiatric Associations Council surveyed senior representatives of the individual EPA member associations (*N* = 44) on the availability, acceptance, financing, and perceived advantages and disadvantages of synchronous telemedicine appointments. The survey was conducted between February and May 2022. Then, 35 of the 44 invited countries provided data. Respondents were satisfied with the accessibility and technical suitability of telepsychiatry for psychiatrists (57.14 and 51.43% rated it excellent and good, respectively), but not with the rate of financial reimbursement (28.57% excellent or good vs 40% poor and very poor). Furthermore, the legislative regulation was considered unsatisfactory (20% excellent or good vs 48.57% poor and very poor).

The task force of the European Psychiatric Association’s (EPA’s) National Psychiatric Associations Council recently surveyed representatives of the individual EPA member associations on the availability, acceptance, financing, and perceived advantages and disadvantages of synchronous telemedicine appointments (Figure 1; results of the survey are presented at https://www.europsy.net/surveys-of-npas/). The survey indicated that the pace of digitalisation of healthcare processes has accelerated during the COVID-19 pandemic across Europe, but that significant technical, financial, ethical–legal, regulatory, and cultural barriers remain to the wide-scale adoption of digital services. Most importantly, respondents voiced concerns regarding potential disadvantages of remote appointments compared to in-person care, lack of technical competency, and barriers to the integration of digital and non-digital services.

The aim of this work is to help mental health specialists and policymakers to navigate the quickly expanding digital mental health space, and thereby to promote and accelerate the clinician- and patient-centred digital transformation of mental health care. To this end, it first

1) discusses the *status quo*, potential, and current challenges of *digital mental health interventions* (DMHIs) and *digital phenotyping*, two major domains of digitalisation, and then

2) provides practical recommendations for the digitalisation of mental health services.

### Methods

The EPA Digital Task Force was established by the EPA President through direct invitation of experts of digital psychiatry as well as EPA members with interest in the topic. Members of the Task Force performed literature searches of the Medline/PubMed Databases and identified relevant works discussing DMHIs and digital phenotyping. Practical recommendations were developed as expert consensus during the regular meetings of the Digital Task Force.

## The *status quo*, potential, and challenges of digitalising mental health care

Digital Health Technologies (DHTs) are software solutions which enable the generation, collection, storage, synthesis, and exchange of health-related information and knowledge in the digital space. They offer the opportunity to partially move the phenotyping, diagnostics, and treatment of mental health disorders to the direct proximity of patients. DHTs like mobile health (mHealth) applications, internet-based technologies, and electronic health records are already used in routine mental health practice. However, their more widespread implementation, the development of new DHT modalities, and their integration with modern data analytic tools are expected to open new possibilities for evidence-based clinical care and personalised and value-based mental health care provision.

In this section, we review two major applications of DHTs, DMHIs (i.e., treatment provision) and *digital phenotyping* (i.e., the generation and collection of disease and treatment-related patient data).

### Digital mental health interventions

Mental health provision is especially well suited for digital platforms: there is a shortage of specialised care, especially in remote rural areas; in many cases, there is no need for in-person examinations; and due to the stigma still associated with mental health disorders, some patients might even prefer the relative anonymity of online visits to crowded waiting rooms [4]. Over the past 25 years, various DMHIs have been developed, providing accessible and acceptable alternatives to traditional face-to-face therapies. In recent years, the number of controlled trials on DMHIs has grown faster than trials on psychotherapy in general. As of 2022, over 300.000 DMHIs offering psychoeducation and treatment for patients with mental health problems have been developed [5, 6]. DMHIs can be grouped as synchronous, asynchronous, and self-guided based on the mode and intensity of interaction with the therapists.

#### Synchronous DMHIs

Synchronous DMHIs (i.e., telepsychiatry) offer treatment as usual via a two-way video or phone call. The adoption of synchronous DHMIs has long been recommended to improve mental health care access and quality. However, only the social distancing measures during the Covid-19 pandemic created a strong policy push to integrate telepsychiatry into standard clinical care [7]. Many patients and clinicians had their first teleconsultation during the pandemic and they have quickly learned to appreciate the convenience of attending a medical appointment from the security and comfort of their home environment. Research conducted before and during the pandemic suggests that telepsychiatry improves access to professional mental health care and outcomes (e.g., reduces emergency visits and hospitalisations), resulting in high cost and time savings and life quality gains [8]. Furthermore, there is increasing evidence that telepsychiatry is a good substitution for face-to-face delivery in terms of effectiveness, care quality, treatment outcomes, and compliance for many mental health problems across a wide range of populations and settings, although long-term real-life evidence is still missing [9, 10].

#### Asynchronous guided DMHIs

Asynchronous (i.e., non-real-time) guided DMHIs use messaging and app notifications to provide feedback and encourage patients to take their medications or complete therapy and daily life tasks. Thus, asynchronous therapy decouples the provider and patient interaction, removing geographic, and temporal boundaries [11, 12]. Finally, mHealth applications and web- and computer-based programs enable *completely self-guided* therapies, participation in peer support groups, journaling, and other symptom assessment and management methods [11]. Asynchronous-guided and self-guided DMHIs enable care delivery at low incremental costs, are easily scalable, and can therefore increase access to evidence-based treatment [11]. These therapy forms require secure, modular software platforms, hosting treatment, and educational materials, providing questionnaires to monitor symptoms and therapy progress, and enabling interactions with the patient [6].

Over the past two decades, approximately 300 controlled trials have been conducted to investigate the efficacy of DMHIs, with internet-delivered cognitive behavioural therapy (iCBT) being the most studied [6]. However, digital versions of other established psychotherapy approaches, like acceptance and commitment or psychodynamic therapy, interpersonal psychotherapy, and mindfulness, as well as the utilisation of augmented and virtual reality for treatment purposes, are also being investigated [6, 13].

Most studies focused on using DMHIs to manage anxiety-, mood-, and trauma-related disorders [11]. According to a recent meta-analysis of 66 RCTs, both guided and self-guided mHealth applications outperformed control conditions for treating depressive, generalised anxiety, and social anxiety symptoms but not for panic or post-traumatic stress symptoms and negative affect [14]. Moreover, a Cochrane review and a recent meta-analysis suggest that guided iCBT is non-inferior to face-to-face CBT for treating anxiety disorders [15, 16]. Interestingly, specific DMHIs components, like behavioural activation, and delivery formats, such as guided therapy (e.g., phone calls and personalised feedback), appear more helpful and effective [17, 18]. Since most of the evidence on the utility of DMHIs for the management of affective disorders is based on patients with mild-to-moderate symptom severity, their applicability for the treatment of severe affective disorders is unclear and should be the focus of future research before these treatments find their way into standard clinical care [19].

The number and quality of studies available on DMHIs for managing the symptoms of schizophrenia spectrum disorders are significantly lower. Although a systematic review from 2014 concluded that such interventions were feasible, results on efficacy for improving medication adherence and managing psychotic symptoms were heterogeneous [20]. A more recent systematic review and meta-analysis found suggestive evidence for the effectiveness of DMHIs using immersive avatar technology but not for other types of DMHIs [21]. RCTs, which have been conducted in recent years to investigate the effect of interventions like text messages, help from lay supporters, cognitive training, and optional synchronous digital appointments on re-hospitalisation, medication adherence, and intensity of hallucinations, have also yielded inconclusive findings [22–24]. However, the heterogeneity of studies regarding treatment settings, interventions, and outcome measures impedes an overall evaluation of the therapeutic potential of DMHIs [25]. There are also promising findings from studies investigating other mental health disorders, like bipolar disorder [26], eating disorders, obsessive-compulsive disorders [27], tic disorders [28], autism spectrum disorder [29], or substance use disorders. However, the evidence for clinical and cost-effectiveness is less abundant for these conditions, which should be taken into consideration before deciding on the clinical deployment of DMHIs for these conditions.

Overall, asynchronous guided and self-guided DMHIs have small-to-moderate effects on alleviating symptoms of depression and anxiety, and human interactions appear to be important determinants of therapy success. Guided DMHIs were more likely to alleviate symptoms and were comparable in effectiveness to face-to-face therapies [11, 17, 30–32].

Despite these promising findings, the failure of DMHIs to engage users in regular therapeutic activities is a major barrier to their successful implementation in clinical practice [33]. According to a meta-analysis of RCTs, 40% of patients receiving unguided psychotherapy dropped out before completing 25% of the planned treatment modules, and only 17% completed all modules [34]. Engagement in real-world settings was even poorer: the 15-day retention rate for mental health apps was only 3.9% [35]. These rates are worrying, given that better engagement has been associated with greater post-interventional mental health improvements [36]. Patient engagement was positively associated with user-friendly, customisable, and technically stable DMHI design; human support and automated reminders; personalisation of the intervention (e.g., metrics, visualisation based on user data); lower costs of the app; and social and gamification features (e.g., levels, reward systems, social characters, contests) [18, 37]. Lack of user technical competence and experience with mHealth applications, low education level and health literacy, low expectations of the intervention, and low trust in the therapists correlated with low adherence rates [37].

### Digital phenotyping

The collection of behavioural phenotypes is essential for diagnosing, managing, and treating mental health disorders. Psychiatry typically deals with symptoms arising in daily life, often depending on contextual factors. The assessment of these symptoms has, however, been traditionally performed indirectly and outside the patients’ daily environment using paper-based clinician-rated measures or self-reports. Consequently, the quality of the generated data depends on clinicians’ training and prior experience and is often influenced by recall biases. In contrast, ecological psychologists have long argued that an in-depth understanding of experiences and behaviour requires longitudinal investigation under real-world circumstances. However, the clinical implementation of such real-life assessments has been traditionally hindered by various technical, attitudinal, and feasibility barriers [38].

Some of these challenges may be overcome by using digital solutions. Indeed, the recent availability of DHTs, such as wearables and a wide array of ambient sensors, coupled with the ubiquitous use of smartphones, opened the door to an unprecedented flow of digital, real-world, and real-time patient data [39]. With the help of DHTs, data can be generated either actively (data entry using specific software or through posting and browsing) or passively (i.e., monitoring of user interactions and sensors in smartphones, wearables, or other smart devices positioned in the environment) [40–42]. Such connected sensor technologies allow frequent, if not continuous, monitoring of human physiology and interaction with the environment with little or no burden for patients, yielding more reliable, valid, and meaningful data. Integrating this constant stream of patient-generated digital phenotypes with one-off clinical measurements could aid decision-making in routine care, research, and public health, for example, by helping set personal baselines for early disease detection; detecting transitions in the course of a disorder (e.g., switches from a high-risk state to psychosis); predicting relapse (e.g., loss of control over drug intake); or by enabling the stratification of homogenous patient groups for clinical research [43]. Furthermore, personalised feedback based on digitally derived patterns of positive affect has been investigated in a randomised controlled trial as a therapeutic tool to reduce depressive symptomatology [44].

However, research on Ecological Momentary Assessment (EMA; also known as experience sampling) methods exemplifies that digital phenotyping introduces specific challenges. Digital EMAs actively sample thoughts, emotions, behaviour, or symptoms through repeated daily surveys. This form of assessment is meant to minimise recall bias, ensure the generalisability of findings to real-world settings (ecological validity), and allow the study of “micro-processes” that may influence behaviour in real-world contexts. However, due to missing consensus about the mode and frequency of surveys, the lack of stringently validated item batteries, and open statistical questions like the handling and interpretation of missing values, EMAs have not yet spread outside the psychological research or the institutional benchmarking contexts [45–47] Moreover, many possible applications of EMA address relatively infrequent clinical events that are not easy detect in the timeframe of clinical trials. In a rare example of a randomised controlled trial in a clinical setting, Faurholt-Jepsen et al. failed to show an effect of smartphone-based monitoring compared to standard treatment on depressive and manic symptoms in patients with bipolar disorder [48]. Thus, researchers have argued that controlled studies with longer assessment periods are needed to explore the full potential of EMA applications [49].

In general, there is a need for systematic studies on the utility of different digital data modalities (e.g., EMA surveys, mobile sensing, sociodemographic data) for specific clinical and research applications.

Nonetheless, first evidence suggests that EMAs could increase precision in the characterisation of at-risk populations [50], in detecting stressful experiences at the individual level [51] and in managing major psychiatric disorders, such as depression [44] or schizophrenic psychoses [52]. An especially promising approach could be to utilise digital phenotypes for machine-learning-based prediction models in order to define group and individual prognostic trajectories better, to take into account a wider range of influencing factors, to optimise treatments, and to more reliably predict clinical outcomes [53].

## Discussion

Digitalisation represents a cultural change in mental health care provision: DHTs and changing customer preferences impact communication channels between mental health workers and patients. We expect that the number and relevance of asynchronous interactions such as communication via email, the management and evaluation of the data originating from patients’ digital devices or other providers will continuously and significantly increase in the future.

Enforcing and incentivising the usage of evidence-based synchronous, asynchronous, and self-guided therapeutic interactions is imperative for establishing a digital and, patient-centred mental healthcare system. Therefore, financial incentives should be aligned with the health benefits achievable through these technologies. This requires sophisticated billing models, that reward healthcare workers for managing asynchronous DHMIs or spending time with the case between synchronous visits, for example, by paying for case-related activities within a time window around synchronous visits or reimbursing based on the generated value (i.e., patient outcomes and/or cost reduction) [54].

To speed up the clinical translation and ensure the long-term real-life utilisation of the technological innovations, DMHIs, DHTs, and digitalisation initiatives must be designed in close interaction with stakeholders, tailored to the needs of patients and therapists and, aligned with clinical workflows [3]. An example for the potential integration of DMHIs in clinical care is stepped-care, where, for example, DMHIs can be offered as a prequel to face-to-face meetings to shorten frustrating waiting times for frontline evidence-based intervention and foster speedy symptom alleviation [54–56]. Or, to ensure that a large number of individuals receive adequate therapy at favourable costs, validated, low-intensity self-guided DMHIs could be offered to individuals with mild-to-moderate symptom severity, whereas resource-intensive, guided or other high-intensity interventions, which benefits were more substantial in individuals with moderate to severe depression, could be saved for individuals at highest need [17, 57]. Furthermore, DMHIs might also be used in aftercare to improve continuity of care, but more evidence is needed on their effectiveness in maintaining treatment gains [58]. It is, however, important not to compromise on the quality of mental health care provision even during the digital transformation of mental health services. Therefore, we recommend that DMHIs should only be included into routine clinical care only if they pass a after a controlled, scientific evaluation process for a specific patient population. Adhering to this will require more evidence, including larger quantities and higher quality data on the utility, safety, and effectiveness of DMHIs and, just as with other forms of therapies, the conduction of systematic, standardised, and transparent efficacy and safety studies. To foster this, we recommend the development of a mental-health-specific DHT and DMHI certification processes.

Modern dynamic data and knowledge repositories, which enable the integration of DMHIs, and digital phenotypes and knowledge generated along the patient’s healthcare journey, are also essential for successfully digitalising mental health provision. These platforms should be designed in a way so that they 1) make data entry and retrieval easy; 2) can be easily enriched with procedural metadata, information originating from patient-owned DHTs, and other external medical and non-medical sources; 3) make the generated information actionable, for example, by providing automated screening instruments for patients at-risk and alerts and reminders for both the clinicians and the patients; and thereby 4) nudge clinicians to deliver care according to guidelines and quality recommendations [59]. If outfitted with modern data analytic tools, these repositories will be able to analyse, synthesise, and present the data on the fly, enabling dynamic knowledge generation and real-time clinical decision support.

To enable sharing of the generated patient data and knowledge (e.g., within the planned European Health Data Space [60]) the collected information must be arranged in a predefined and internationally harmonised structure, like the Fast Health Interoperability Resources format and mapped onto international medical terminologies standards, like SNOMED-CT (clinical terminology), ICD (diagnoses), and RxNorm (medications). Therefore, minimum data structure and interoperability standards must be established across all mental health providers.

Real-world data containing highly sensitive personal information must be handled securely in compliance with data protection frameworks, such as the General Data Protection Regulation of the European Union (GDPR) and national laws. Information and consent forms must be standardised to allow data exchange and use across healthcare providers. For example, the Broad Consent, which the German Medical Informatics Initiative developed, fulfils the requirements of all German research ethics committees and data protection authorities, and thus establishes the necessary ethical- and legal environment for large-scale data collection and exchange [61, 62]. Similar initiatives could be developed for other countries as well to enable sharing data in the European Health Data Space.

Finally, although DHTs and DMHIs empower patients to exercise greater choice and more control of their treatment, navigating this digital space also comes with new responsibilities and may require acquiring new knowledge and technical skills besides access to the appropriate hardware and software. Individuals not digitally versed, or not having the access to the necessary digital devices, for example, elderly patients or patients of lower sociodemographic status, might be left behind. Therefore, to create equal chances for all stakeholders to benefit from digitalisation and foster communication within a multi-professional team, we recommend 1) the identification of the minimal technical requirements (devices, connectivity, technical support); 2) the development of educational resources for both members of the health care teams and patients (IT-knowledge and data security); 3) the establishment of the financial means and legal environment to ensure equal access to the minimally required hardware and software solutions for all service users; 4) the systematic measurement of the digital skills of patients and health care professionals; 5) the development of guidelines on using DHTs and DMHIs; and 6) the establishment of new professional roles, like that of a data steward, who can educate and guide patients in the digital space [63].

In summary, there is increasing evidence that DMHI can augment or (temporarily) even substitute face-to-face mental health provision regarding outcomes, compliance, and access to services. However, the uptake of DMHI and the digitalisation of mental health services still need improvement. Changing this will require a systematic assessment of the status quo and consensus-based – that is, stakeholder (health professionals, developers, patients, users, and policymakers) backed – system-wide adjustments on both the macro (e.g., regulatory and legal framework, financing) and micro (e.g., infrastructure, workflow, training, and culture) level. To foster this process and thus the successful and sustainable digitalisation of mental health services and research, the EPA expert panel has identified, relying on previous recommendations, the following action points and priority areas (Table 1) [64, 65].

**Table 1.** Key recommendations of the European Psychiatric Association for the digitalisation of mental health services

Recommendation	Aims
Establish a shared legal, ethical, and regulatory framework on digital health technologies	Ensure legal clarity and ethical correctness Safeguard human rights, privacy, and data security Integrate DMHIs and DHTs into established treatment processes Ensure the quality of offered digital interventions
Develop adequate financing strategies for digital care provided by mental health professionals	Guarantee the financial viability of digital mental health care Foster adoption and further development of DMHIs and DHTs Guarantee equal access to DMHIs and DHTs to all service users
Establish dynamic data and knowledge repositories	Ensure the integration of real-life (e.g., electronic health records, patient owned DHTs) and research information Make the generated information actionable Nudge mental health workers to deliver care according to guidelines
Develop standards that ensure the interoperability and usability of health technology	Facilitate GDPR-conform data sharing Facilitate the development and sharing of best practices
Enhance digital health literacy and skills in the public and the mental health workforce	Increase awareness and acceptance of digital mental health products and services Make stakeholders aware of the benefits and pitfalls of new technologies, like artificial intelligence biases Foster trust in digital tools in mental health care and prevention efforts
Stimulate and fund research into digital mental health	Systematically measure the effectiveness, real-life utility and tolerability of DHTs and DMHIs Assess the implementation process of digital mental health initiatives Provide public funding for development-independent studies into the effectiveness and safety of DHTs and DMHIs
Establish a mental health specific certification process	Ensure that DMHIs and DHTs are delivered in accordance with best practices and up to date evidence Ensure that DMHIs, DHTs and digitalisation initiatives align with the clinical workflows
